# Calcineurin-dependent regulation of gap junction conductance and connexin phosphorylation in guinea pig left atrium

**DOI:** 10.1007/s00424-023-02798-9

**Published:** 2023-03-14

**Authors:** R. I. Jabr, S. C. Salvage, F. S. Hatch, C. H. Fry

**Affiliations:** 1 Department of Neuroscience, Physiology & Pharmacology, University College London, London, UK; 2 Department of Biochemistry, University of Cambridge, Cambridge, UK; 3 Department of Biochemistry and Physiology, Surrey University, Guildford, UK; 4 School of Physiology, Pharmacology & Neuroscience, University of Bristol, Biomedical Sciences Building, University Walk, Bristol, BS8 1TD UK

**Keywords:** Left atrium, Gap junctions, Gap junction electrical conductance, Calcineurin, Phosphorylation, Connexin

## Abstract

Atrial fibrillation (AF) occurs from disordered atrial action potential conduction and is associated with reduced gap junction electrical conductance (*G*_j_). The Ca^2+^ and calmodulin-dependent phosphatase, calcineurin, reduces *G*_j_ in ventricular myocardium *via* a protein phosphatase-1 (PP1)-dependent pathway culminating in phosphorylation of serine368 on connexin43 (pSer368-Cx43). However, characterisation of corresponding pathways in left atrial myocardium, which have a more complex connexin subtype profile, is undefined and was the aim of this study. *G*_j_ was measured in guinea-pig left atrium from the frequency-dependent variation of intracellular impedance; intracellular [Ca^2+^], ([Ca^2+^]_i_) in low-Na solution was measured by Fura-2 fluorescence. Phosphorylation of guinea-pig Ser368-Cx43 residues was measured by Western blot; Cx40 was immunoprecipitated and probed for serine/threonine residue phosphorylation. Low-Na solution reversibly reduced *G*_j_, in turn attenuated or prevented by calcineurin inhibitors cyclosporin-A or CAIP, respectively. Moreover, Ser368-Cx43 phosphorylation in low-Na solution was also prevented by CAIP. Changes were partially prevented by fostreicin (FST), a protein phosphatase-2A (PP2A) inhibitor; but not by tautomycin, a PP1 inhibitor. Serine/threonine residues on Cx40 were also phosphorylated in low-Na solution; prevented by CAIP and attenuated by FST. Reduced *G*_j_ with raised [Ca^2+^]_i_ is paralleled by a changed Cx43/Cx40 phosphorylation status; changes mediated by calcineurin and PP2A-dependent pathways, but not PP1. The pharmacological profile underlying changes to guinea-pig atrial gap junction electrical conductance with raised intracellular [Ca^2+^]_i_ is fundamentally different from that in ventricular myocardium. This provides a targeted drug model whereby atrial and ventricular myocardium can be selectively targeted to correct conduction defects.

**Supplementary Information:**

The online version contains supplementary material available at 10.1007/s00424-023-02798-9.

## Introduction

Atrial fibrillation (AF) remains a major cause of morbidity and mortality, including risk of thromboembolism and cerebrovascular stroke [1]. A rise of the sarcoplasmic [Ca^2+^] ([Ca^2+^]_i_) is a key contributor to initiation of AF [2] and consequential generation of re-entrant rhythms that require a region of slow action potential (AP) propagation. The pathway whereby raised [Ca^2+^]_i_ reduces AP propagation is less clear, although a key factor is the electrical conductance (*G*_j_) offered by gap junctions between contiguous myocytes [3]. Gap junctions (GJ) in atrial myocardium are composed of connexin (Cx) phosphoproteins, with two main isoforms, Cx40 and Cx43, that form homotypic or heterotypic structures, each with a characteristic unit value of *G*_j_ [4]. Overall *G*_j_ and AP propagation velocity will depend on the relative expression of Cx isoforms [5, 6].

Moreover, the phosphorylation state of several serine (Ser) and threonine (Thr) residues on Cx isoforms determines unit *G*_j_ and the role of Ser/Thr protein kinases (PK) and phosphatases (PP) are crucial [7, 8]. Ca^2+^/calmodulin-dependent PKII (CaMKII) phosphorylation targets on Cx43 have been extensively mapped [9]. However, only serine-368 (Ser368) Cx43 phosphorylation, targeted by PKC and which decreases *G*_j_, has been characterised [10]. Access to the Ser368-Cx43 residue is by dephosphorylation of a gatekeeper residue Ser365-Cx43 [11].

Under physiological conditions Ser365-Cx43 is in a phosphorylated state (pSer365-Cx43) and Ser368-Cx43 is poorly phosphorylated to maintain a relatively high *G*_j_. We showed in guinea-pig ventricular myocardium [12] that raised [Ca^2+^]_i_ is accompanied by slowed action potential conduction and reduced *G*_j_, accompanied by relative dephosphorylation of Ser365-Cx43 as well as phosphorylation of Ser368-Cx43. The changed phosphorylation state of Ser365-Cx43 is dependent on the activity of the Ca^2+^/CaM-dependent Ser/Thr PP, calcineurin (Cn), with subsequent PKC-dependent Ser368-Cx43 phosphorylation [12]. The action of calcineurin to initiate this process is indirect, *via* its activation of the Ca^2+^-independent phosphatase PP1. However, there was no functional role for the Ca^2+^-independent phosphatase PP2A.

With atrial myocardium less is known about the Ca^2+^-dependent regulation of *G*_j_ through changes to Cx phosphorylation and the role of Cn. Moreover, there is an additional complication of two predominant atrial Cx isoforms, Cx43 and Cx40. Less is known about the relationship between phosphorylation sites and electrical conductance of Cx40 gap junctions although a potential role is suggested from changes to Cx40 homotypic *G*_*j*_ by raised intracellular cAMP or β-adrenoreceptor activation [13, 14].

This study aimed to answer the following questions in left atrial myocardium: does raised [Ca^2+^]_i_ reduce *G*_j_; is there a role for calcineurin in any change to *G*_j_; what are the contributions of PP1 and PP2A to any calcineurin-dependent pathway; and are there phosphorylation changes to Cx43 and Cx40 that accompany changes to *G*_j_. Phosphorylation status of Cx43 was assayed as the relative changes at Ser368; however, as sites affecting *G*_j_ on Cx40 are less well understood changes to total serine or threonine phosphorylation were measured.

## Methods

### Tissue sources and ethics

Dunkin-Hartley male guinea-pigs (300–400 g) were heparinised (100 units, i.p.), euthanised by cervical dislocation—confirmed by absent corneal and spinal reflexes—the heart excised and the left atrium dissected in Tyrode’s solution. Experimental protocols adhered to ARRIVE guidelines [15]; begun at Surrey University and finalised at the University of Bristol. Animals were procured by the local animal services units, housed in straw-floored plastic cages at 22 °C with a 12-h light–dark cycle and with water and food available *ad libitum*.

### Measurement of gap junction conductance

Left atrial strips (< 1.0 mm diam, ≈5 mm length) were dissected immediately after atrial excision and mounted in a three-chamber Perspex bath at 37 °C. Chambers were separated by thin rubber membranes and the strip pulled gently through holes in the membranes so as to span the three chambers. Outer chambers were superfused with Tyrode’s and the central chamber filled with mineral oil. Alternating current (a.c.; 0.1–100 kHz; 10 mV peak-to-peak) was passed between the two outer chambers via Pt-black electrodes. This experimental system constrained a.c. to pass through the intracellular pathway of the preparation, with a small parallel resistance shunt, *r*_ec_, from Tyrode’s solution trapped in the extracellular space. This system formed one arm of an a.c. (Wien) Bridge arrangement (Wayne-Kerr 6425, Bognor Regis, UK) to allow total impedance (*z*) to be recorded as a function of a.c. frequency. The method and its validation have been described in detail [5, 16, 17]. Pt-black electrode impedance, *z*_elec_, in series with the preparation in the oil gap, was measured separately in a large volume of Tyrode’s solution; extracellular fluid resistance, *r*_ec_, in parallel to total intracellular impedance (*z*_i_), was also measured separately as the resistance between two needle Pt-black electrodes placed in the muscle within the central oil gap. Subtraction of *z*_elec_ and *r*_ec_ from total impedance, *z*, yielded a value of left atrial *z*_i_ over this range of frequencies. Values of *z*_i_ were transformed to yield a frequency-independent sarcoplasmic conductance (*g*_c_), and an in-series frequency-dependent gap junction conductance (*g*_j_). Two sets of recordings of *z* were made for each intervention, five minutes apart; values that were within 5% were averaged and retained for analysis. After control recordings in Tyrode’s solution the mineral oil was removed, the preparation exposed to a gassed intervention solution for 20 min before the oil was re-applied and new measurements made. Finally, preparation length and diameter in the oil gap were measured to yield ‘specific’ gap junction conductance, *G*_j_ (mS.cm^−2^) values, i.e. independent of preparation dimensions.

### Histology

Atrial strips as used for impedance measurements were snap-frozen in liquid N_2_, immediately after the heart was removed and dissected (*t* = 0) or after 45 min (*t* = 45) in an oil-gap chamber and stored at –80 °C. Frozen sections (12 μm) were fixed with ice-cold 4% paraformaldehyde (15 min at room temperature) and stained with haematoxylin and eosin. Sections were mounted and viewed at × 40 magnification.

### Measurement of [Ca^2+^]_i_

Large myocyte clusters were prepared by collagenase dispersion of the left atrium and loaded with 5 µM Fura2-AM. [Ca^2+^]_i_ was recorded as *R*_340/380_, the fluorescence emission (410–500 nm) ratio with illumination successively (32 Hz) at 340 or 380 nm. Output data were transformed through a third-order Butterworth band-pass filter centred on 32 Hz.

### Western blotting

Guinea-pig hearts were perfused through the coronary vasculature for 10 min with Tyrode’s or low-Na Tyrode’s solutions in the absence or presence of phosphatase inhibitors. The left atrium was then rapidly snap-frozen in liquid N_2_. A whole-tissue homogenate was prepared using radio-immunoprecipitation assay (RIPA) lysis buffer, supplemented with MS SAFE protease inhibitors (Sigma, UK). Protein (30 μg) from each sample was prepared in NuPAGE® LDS sample buffer 4X with NuPAGE® sample-reducing agent 10X (Invitrogen, UK). Protein samples were resolved by 12% polyacrylamide SDS-PAGE and transferred to PVDF membranes (Invitrogen, UK), blocked with Odyssey blocking buffer (Li-COR Biosciences, Ltd, UK; 2 h; RT) and incubated overnight at 4 °C with primary antibody in 1% BSA-Tris buffered saline-Tween (TBS-T). Membranes were washed with TBS-T and incubated with secondary antibody (1:10000 dilution; 1 h, RT). Protein bands were imaged using an Odyssey infra-red (IR) imaging system (UK); red and green channels. Densitometric analyses used colour filters on Image-J software. Cx43 generally appeared as several separate bands (see Fig. 2C) and the whole set of bands were included in the densiometric analysis for total Cx43 (T-Cx43). Band densities of phosphorylated s368-Cx43 protein were normalised to total Cx43 (T-Cx43) densities and T-Cx43 normalised to GAPDH. Immunoprecipitated Cx40 (IP-Cx40. below) was probed for serine/threonine phosphorylation and band densities were also normalised to their corresponding T-Cx40 values.

### Immunoprecipitation

This was undertaken to analyse the phosphorylation profile of Cx40 under different interventions, as antibodies to specific phosphorylated residues were unavailable, as they were available for Cx43 (e.g. phosphorylated serine368-Cx43). Protein G Sepharose 4 Fast Flow beads (25–50 µl bead slurry; binding capacity ≥ 20 µg IgG/µl slurry; GE Healthcare Life Sciences, UK) were conjugated with Cx antibodies (10 µl, containing 2 µg IgG) then washed with ice-cold phosphate-buffered saline (PBS) to remove unbound antibodies, as per supplier’s protocol. A protein aliquot (100 µg) from a frozen tissue sample was added to the conjugated beads and incubated overnight at 4 °C. Unbound protein was extracted and washed with PBS to remove residual protein. Beads were then incubated (95°C; 5 min) with NuPAGE® LDS sample buffer 4X and NuPAGE® sample-reducing agent 10x. After centrifugation, unbound and bound samples were processed by Western blot for total-Cx40, or phosphoserine/ phosphothreonine protein; the latter were normalised to total-Cx40 values.

### Solutions and antibodies

Tyrode’s solution contained (mM): NaCl 118; KCl 4.0; NaHCO_3_ 24; MgCl_2_ 1.0; CaCl_2_ 1.8; NaH_2_PO_4_ 0.4; glucose 6.1; Na pyruvate 5.0; gassed with 95%O_2_/5%CO_2_, pH 7.40 ± 0.05. Low-Na Tyrode’s (29.4 mM Na), to raise the intracellular [Ca^2+^] ([Ca^2+^]_i_) was similar except that NaCl was replaced by TrisCl (pH to 7.4 with 1 M NaOH). The following inhibitors were used: two Cn inhibitors: i) cyclosporin-A (CysA, 5 µM; 10 mM DMSO stock), ii) Cn autoinhibitory peptide (CAIP, 50 µM; 100 mM aqueous stock) a highly-selective, cell-permeable peptide. Other phosphatase inhibitors were: a PPI inhibitor, tautomycin (TTM, 5 nM; 65.2 µM PBS stock) and a PP2A inhibitor, fostriecin (FST, 100 nM; 20 mM DMSO stock). CaMKII inhibitors were KN-93 (5 µM; 50 mM DMSO stock) and AIP (10 µM; 500 µM aqueous stock). A PKC inhibitor was chelerythrine (2 µM: 10 mM aqueous stock). The gap junction blocker, heptanol [16, 18] was used as a 1 mM solution in Tyrode’s solution. Reagents were from Sigma-Aldrich, UK, except for CysA (Calbiochem, UK), as well as AIP and FST (Tocris, UK).

Primary antibodies were: anti-total Cx43 (1:1000, rabbit polyclonal, Santa Cruz Biotechnology UK, sc-9059); anti-pS368-Cx43 (1:1000, goat polyclonal, Santa Cruz, sc-25165); anti-total Cx40 (1:1000, goat polyclonal Santa Cruz, sc-20466); anti-GAPDH (1:1000, mouse monoclonal, Santa Cruz, sc-365062); anti-phosphoserine (1:1000, mouse monoclonal, Abcam, ab7851); anti-phosphothreonine (1:1000, rabbit polyclonal, Abcam, ab9337). Secondary antibodies (1:10,000 dilution, Li-COR Biosciences Ltd, UK) were: IRDye-680 donkey anti-mouse, IRDye 680 donkey anti-goat, IRDye-800 donkey anti-rabbit and IRDye-800 donkey anti-goat antibodies. Primary antibodies were tested against left ventricle and brain lysates to find optimal dilutions.

### Statistical analyses and curve-fits

Electrophysiological, Ca^2+^ and Western blot data are quoted as means ± SD, except where specifically designated; when several values under a particular condition were collected from a sample, these were averaged before statistical analyses, *n* = number of separate hearts. Comparisons between continuous data sets were tested by two-way ANOVA, with *post hoc* parametric tests. The null hypothesis was rejected at p < 0.05. Statistical tests used Excel and www.vassarstats.net. Impedance locus (R *vs* -X) plots were fitted to the equation of a circle with a radius, *r*, centre displaced, *a*, along the abscissa, (*R*-a)^2^ + (-*X*)^2^ = *r*^2^; where R is resistance and -X is reactance in the complex plane. Non-linear curve-fits used a Levenberg–Marquardt algorithm (KaleidaGraph, Synergy Software, Reading, PA, USA).

## Results

### Tissue viability and intracellular [Ca^2+^], [Ca^2+^]_i_, measurements

Tissue structural integrity over the experimental time-course (up to 40 min) was verified by fixing specimens either immediately after the heart was retrieved (*t* = 0), or after 40 min (*t* = 45) in an impedance chamber and staining with H&E (Supplemental Fig. 1A, *n* = 4 in total). Previous experiments also showed that tissue ATP was maintained over this time frame [18]. With large clusters of myocytes [Ca^2+^]_i_ was raised (increase of Fura-2 R_340/380_ ratio) by low-Na superfusion and sustained for a period similar to the time-course of an experiment to measure *G*_j_ (Supplemental Fig. 1B). The resting R_340/380_ ratio in control was unaffected by cyclosporin-A (5 µM, CysA; *n* = 6), or CAIP (50 µM, *n* = 4). In addition, the increase in low-Na solution was similar in all three conditions (∆R_340/380_ ratio: 0.092 ± 0.030; 0.103 ± 0.015; 0.106 ± 0.008, respectively; p > 0.05, 2-way ANOVA).

### Gap junction conductance, G_j_: effect of low-Na solution; controls

**Fig. 1:**
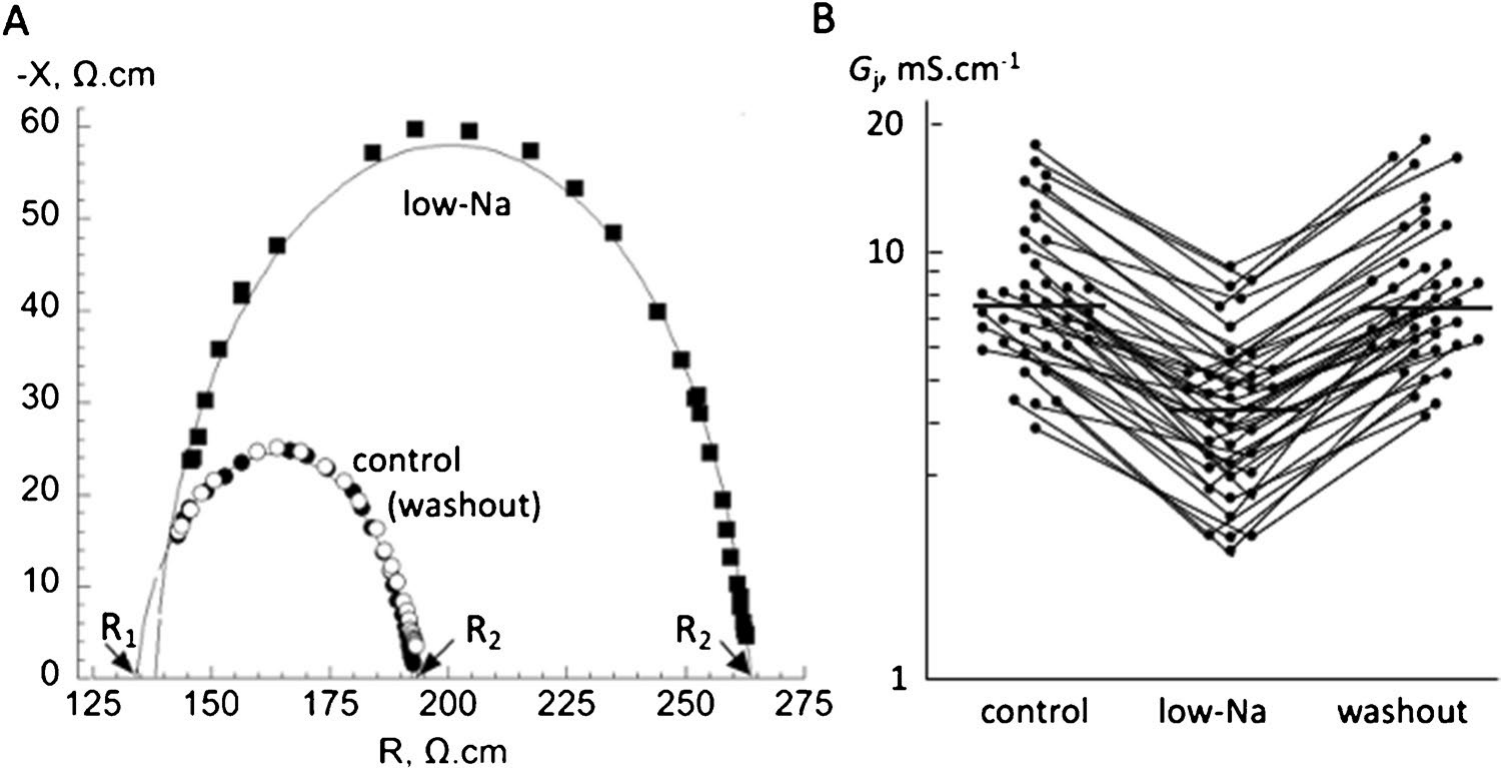
Gap junction electrical properties in control and low-Na solutions. **A** Plots of calculated resistance (*R*) and reactance (-*X*) in the complex plane from an individual experiment in control solution (closed circles); low-Na solution (closed squares); washout to control solution (open circles). Fits to the data are made with a circle function with a centre of radius shifted along the *R*-axis (see Methods). The intercepts with the abscissa, R_1_ and R_2_, are used to estimate sarcoplasmic resistivity, *R*_c_, and intracellular resistivity, *R*_j,_ from which gap junction resistivity, *R*_j,_ is derived. **B** Values of *G*_j_ (= 1/*R*_j_) in control solution, in low-Na solution and after washout to control solution. The connecting bars are data from individual experiments. Horizontal bars in each data set show median values; note the logarithmic scale of the ordinate

Figure 1A shows an example of resistance (R) and reactance (-X) complex plane plots in Tyrode’s solution (closed circles), on exposure to low-Na solution (closed squares) and then on return to Tyrode’s solution (open circles). The intercepts of the circle fits with the abscissa are functions of sarcoplasmic resistivity, *R*_c_ (R_1_) and total intracellular resistivity, *R*_i_ (R_2_): gap junction resistivity, with *R*_j_ as *R*_i_-*R*_c_. Low-Na solution reversibly increased *R*_i_, but not *R*_c_, and hence reversibly increased *R*_j_. Henceforth, *R*_j_ and *R*_c_ values (units Ω.cm) are expressed as inverse specific resistivity or conductance values (*G*_j_, *G*_c_, units mS.cm^−1^).

Low-Na solution consistently and reversibly reduced *G*_j_ to 4.5 ± 1.9 mS.cm^−1^, compared to pre- and post-intervention values of 8.4 ± 3.7, 8.5 ± 3.6 mS.cm^−1^ respectively (*p* < 0.001;* n* = 40, Fig. 1B): these two values in control solution were not significantly different (*p* > 0.05), with the post-intervention value 101.4 ± 8.1% of the pre-intervention value. The percentage reduction of *G*_j_ in low-Na solution was similar between preparations (54.3 ± 11.4% pre-control, *p* < 0.0001) and independent of the initial control value (*r* = 0.025).

Sarcoplasmic electrical conductance (*G*_c_) was similar in Tyrode’s and low-Na solutions: 7.3 ± 2.2 and 6.7 ± 2.5 mS.cm^−1^ (*n* = 40; *p* < 0.05). Values were unaffected by any interventions and are not further reported. In Tyrode’s solution the electrical conductances offered by the sarcoplasm and gap junctions are thus similar – see Discussion for biophysical implications.

Heptanol (1 mM in Tyrode’s) reduces gap junction conductance [16, 18] and was tested in separate preparations. Sarcoplasmic conductance was unaffected by heptanol (*G*_c_ 7.8 ± 1.60 *vs* 7.9 ± 1.57 mS.cm^−1^, *p* > 0.05, *n* = 5; control *vs* heptanol) whereas gap junction conductance was significantly decreased (*G*_j_ 9.2 ± 1.5 *vs* 4.5 ± 0.4 mS.cm^−1^, *p* < 0.01, *n* = 5).

### Gap junction conductance and ser368-Cx43 phosphorylation – role of calcineurin inhibitors

**Fig. 2:**
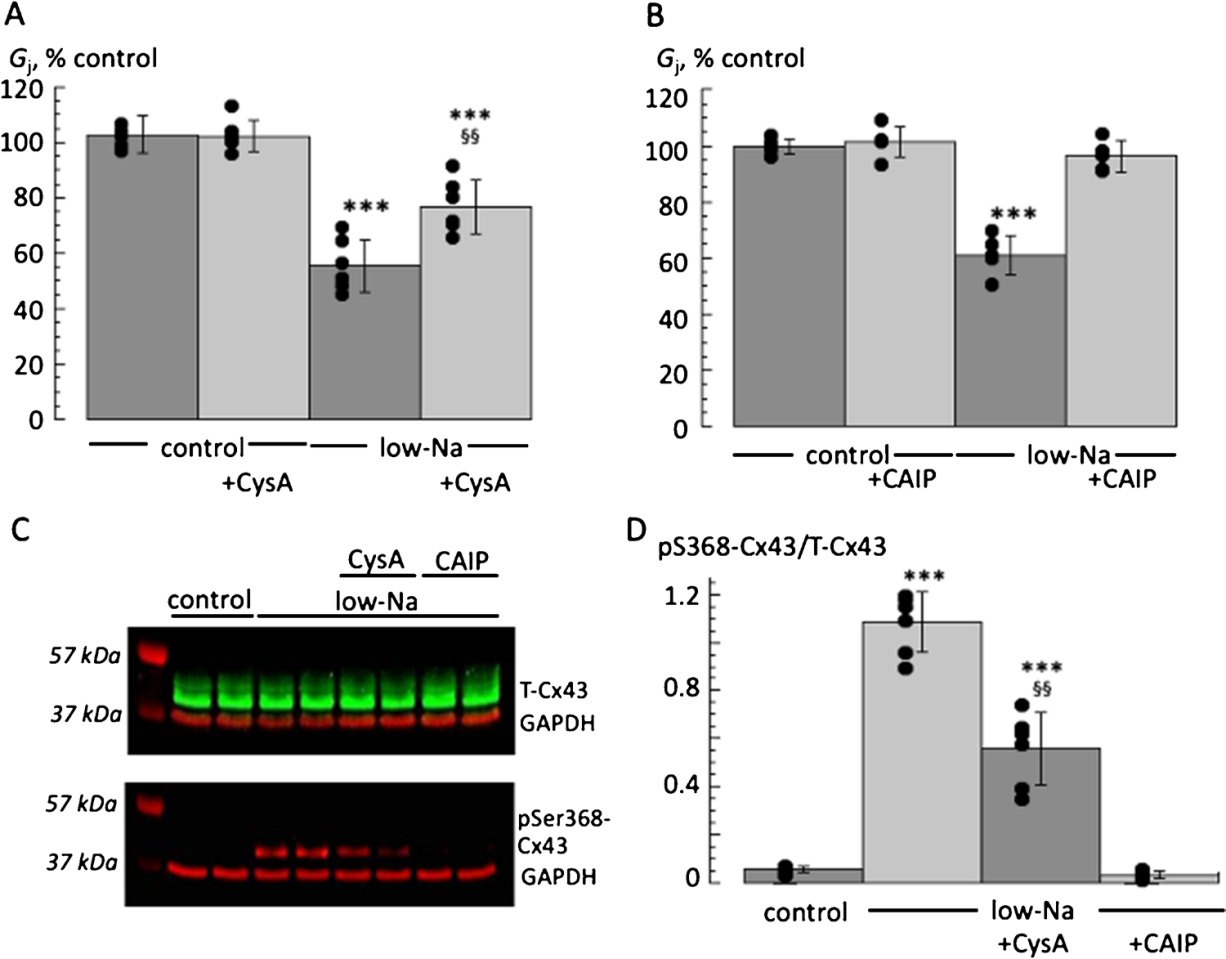
Calcineurin inhibitors (CysA and CAIP) on gap junction conductance, G_j_, and Cx43 phosphorylation. **A** G_j_ values in control or low-Na solution, each in the absence or presence of cyclosporin-A (CysA, *n* = 6). *G*_j_ is expressed as a percentage of values in control solution. **B** As with part A but with the more specific calcineurin inhibitor, CAIP (*n* = 6). **C**: Representative Western blots for total Cx43 (T-Cx43, upper blot) and pSer368-Cx43 expression (lower plot) and also, in each case, GAPDH – see Methods for details. Lane 1 is molecular weight markers; Lanes 2 and 3 are from control tissue derived protein, lanes 4–9 lysates from low-Na treated tissues (lanes 6–7 and 8–9, are also plus CysA and CAIP, respectively). **D:** signal quantitation for pSer368-Cx43, normalised to T-Cx43. Data are mean ± SD, all *n* = 6. ***p < 0.001; low-Na (± calcineurin inhibitor) *vs* control: ^§§^*p* < 0.01, low-Na *vs* low-Na + calcineurin inhibitor

The reduction of *G*_j_ by low-Na solution was significantly attenuated in the presence of CysA (76.9 ± 9.8% of control *vs* 55.5 ± 9.5%; *n* = 6; *p* < 0.01); Fig. 2A, although not fully recovered compared to control levels (p < 0.001). With the more selective calcineurin inhibitor, CAIP, G_j_ was unaffected compared with control levels in low-Na solution (96.2 ± 5.4% of control *vs* 60.9 ± 7.1%; *n* = 6; *p* = 0.001); Fig. 2B.

With tissue from the same hearts, incubation in low-Na solution for 20 min also significantly increased Ser368-Cx43 phosphorylation (pSer368-Cx43; Fig. 2C,D; *p* < 0.001, *n* = 6). pSer368-Cx43 data are presented as normalised to total Cx43 (T-Cx43) expression. T-Cx43 expression in turn was normalised to GAPDH (1.09 ± 0.17 *vs* 1.09 ± 0.07 in control and low-Na solutions, *n* = 8; p > 0.05) and this ratio was unaffected by any interventions used: thus T-Cx43/GAPDH levels are not further reported. In concordance with the *G*_j_ data, the increase of pSer368-Cx43/T-Cx43 expression ratio in low-Na solution remained (*p* < 0.001, *n* = 6), but significantly less so in the presence of CysA (*p* < 0.01, *n* = 6). However, the increase of pSer368-Cx43/T-Cx43 expression ratio was completely prevented by CAIP.

### Gap junction conductance and the role of protein phosphatases PP1 and PP2A

**Fig. 3:**
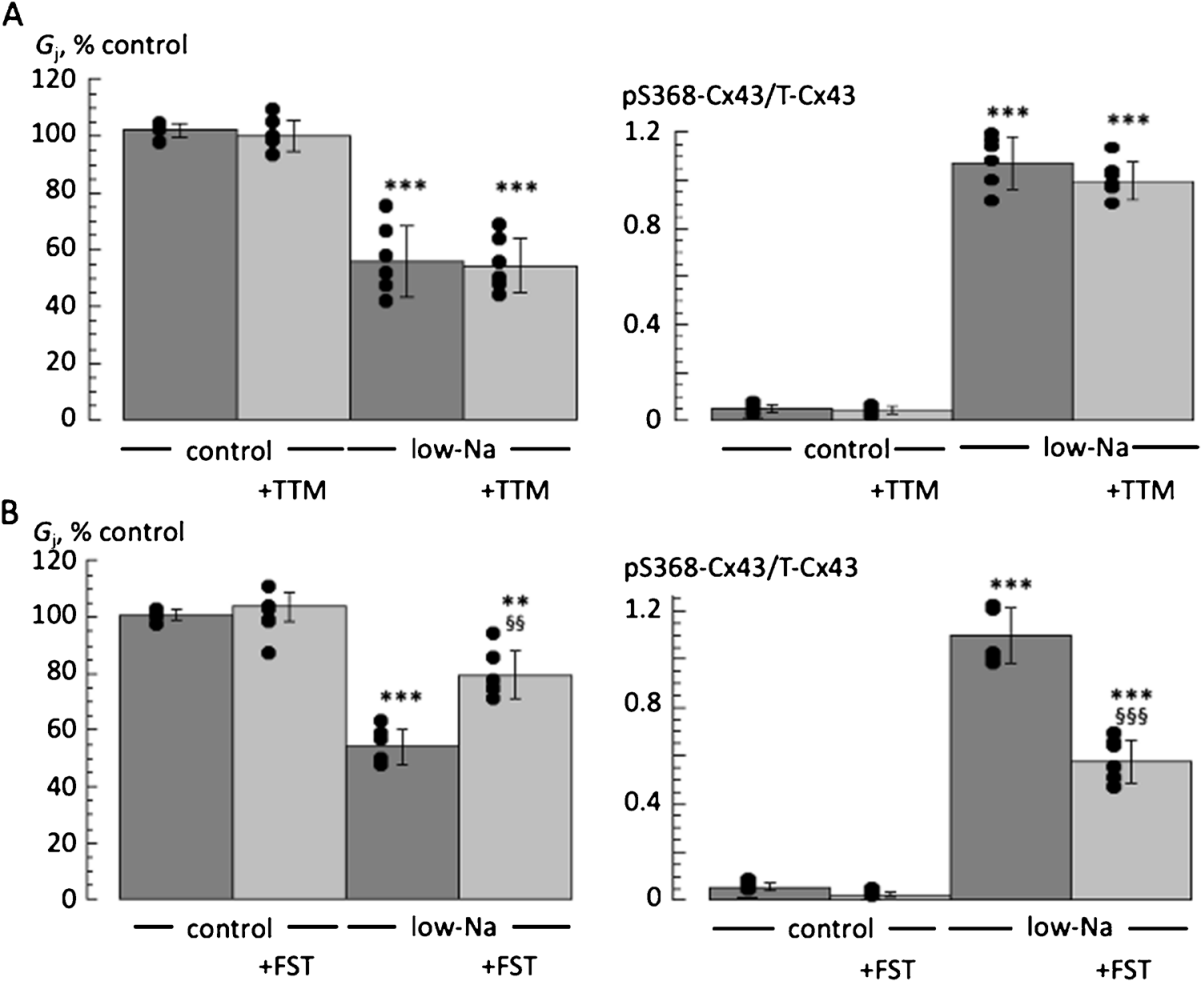
Tautomycin (TTM) and fostriecin (FST) on gap junction conductance, G_j_, and Cx43 phosphorylation in normal and low-Na solution. **A** Effect of TTM. Left; *G*_j_ in low-Na solution as percentage of values in control and in the absence or presence of TTM (*n* = 6, left panel). Right; protein expression of pSer368-Cx43 normalised to total Cx43 (T-Cx43) in control and low-Na solution in the absence or presence of TTM (*n* = 6, right panel). **B** Effect of FST on the same experimental paradigm. ***p < 0.001; low-Na *vs* control: ^§§^*p* < 0.01; ^§§§^*p* < 0.001 low-Na *vs* low-Na + calcineurin inhibitor

The PP1 inhibitor tautomycin (TTM, 5 nM) had no effect on *G*_j_, either in control or in low-Na solution (Fig. 3A, left panel; *n* = 6). These data were paralleled by a lack of effect of TTM on pSer368-Cx43 expression in control or in low-Na solution (Fig. 3A, right panel; *n* = 6). The PP2A inhibitor fostriecin (FST, 100 nM; *n* = 6) also had no effect on *G*_j_ or pSer368-Cx43 levels in control solution (Fig. 3B, left and right panels. respectively). However, in low-Na solution FST partially reversed the reduction of *G*_j_, (*p* < 0.01, *n* = 6), as well as the increase of pSer368-Cx43 expression (*p* < 0.001, *n* = 6). In all interventions T-Cx43 expression remained unchanged with respect to control.

### Gap junction conductance: CaMKII and PKC inhibitors

**Fig. 4:**
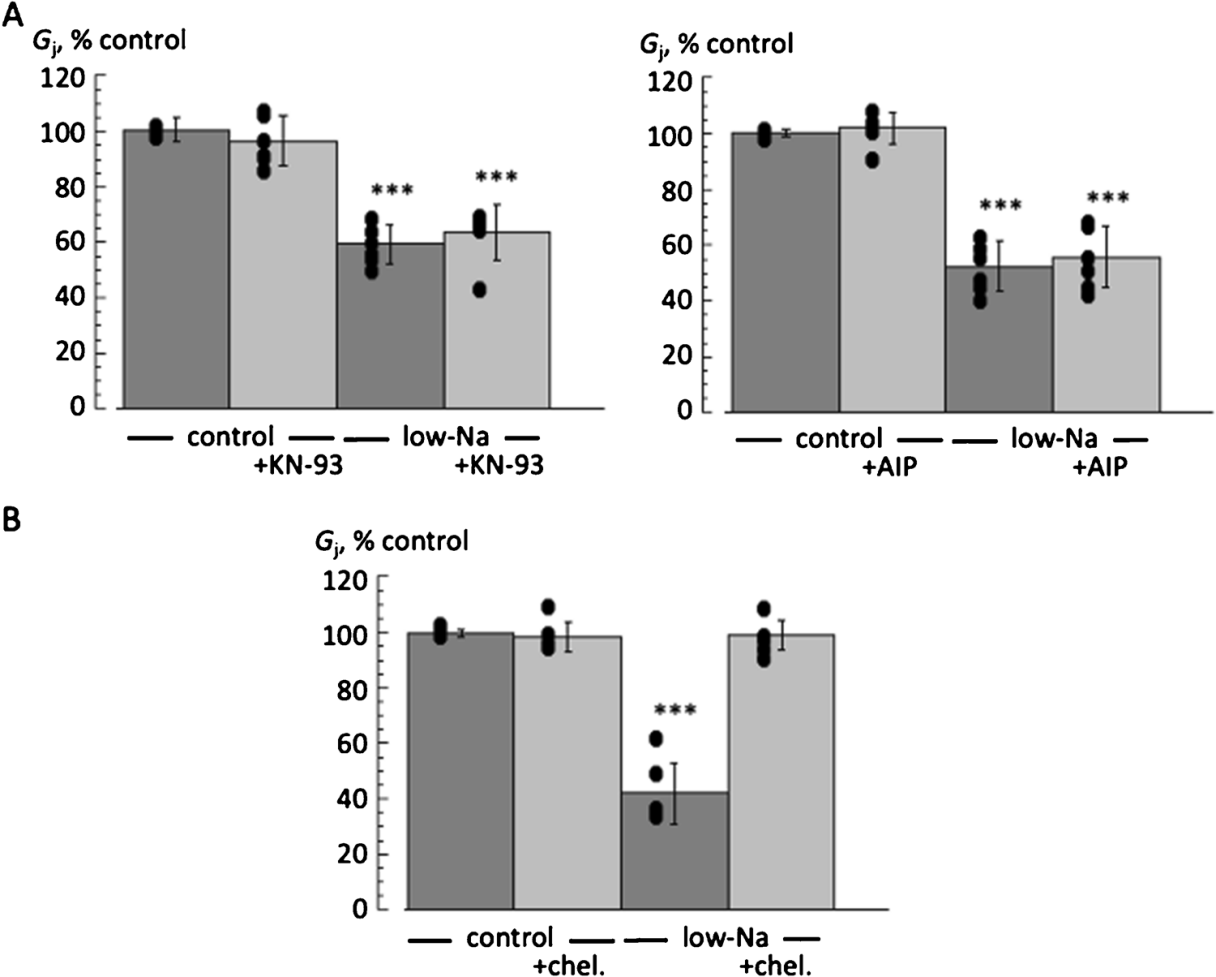
CaMKII and PKC inhibitors on gap junction conductance, G_j_. **A** Effect on *G*_j_ of the CaMKII inhibitors KN93 (left) and AIP (right). In control and low-Na solution (both *n* = 6). **B** Effect on *G*_j_ of the PKC inhibitor chelerythrine (*n* = 5) ***p < 0.001; low-Na *vs* control

The CaMKII inhibitors KN-93 (5 µM) and AIP (10 µM) had no significant effect on *G*_j_, either in control or in low-Na solutions (Fig. 4A). In the absence or presence of KN-93, in low-Na solution *G*_j_ was 59.3 ± 6.8 *vs* 63.7 ± 9.9% of control in low-Na solution (*p* > 0.05, *n* = 6). For AIP experiments, in low-Na solution *G*_j_ was 52.3 ± 8.7 *vs* 55.8 ± 11.0% control in its presence or absence (*p* > 0.05, *n* = 6). Conversely, the PKC inhibitor chelerythrine (2 µM) prevented the decrease of *G*_j_ in low-Na solution (Fig. 4B). In low-Na solution *G*_j_ was reduced to 42.7 ± 10.9% *vs* 99.0 ± 5.5% of control in the absence or presence of chelerythrine, respectively (*p* < 0.01, *n* = 5).

### Phosphorylation of IP-Cx40 in low-Na solution, the calcineurin pathway

**Fig. 5:**
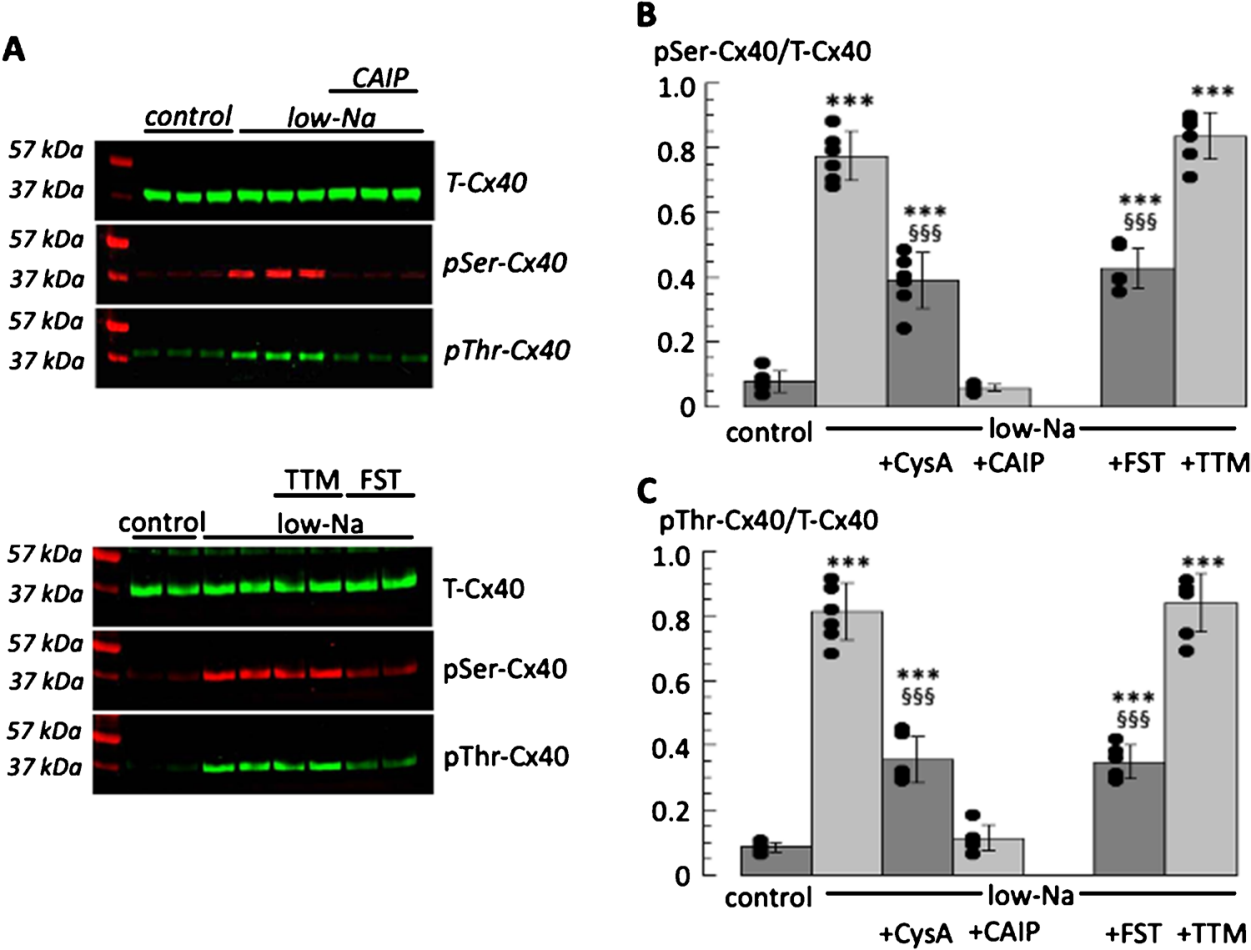
Phosphorylation of serine and threonine Cx40 residues; effects of calcineurin and phosphatase inhibitors. **A** Representative Western blots for the detection of T-Cx40, pSer-Cx40 and pThr-Cx40 phosphorylation. Upper panel set: Lane 1, molecular weight markers; lanes 2–4, control tissue, lanes 5–10, low-Na treated tissues (lanes 8–10 also treated with CAIP). Lower panel set: Lane 1, molecular weight markers; lanes 2,3 control tissue, lanes 5–9, low-Na treated tissues (lanes 7,8 and 9,10 also treated with TTM and FST respectively). **B** Signal quantitation of pSer-Cx40 data normalised to T-Cx40 for control and low-Na data (in the additional presence of CysA, CAIP, FST and TTM). **C**: Signal quantitation of pThr-Cx40 data normalised to T-Cx40 as in part B. ***p < 0.001; low-Na *vs* control: ^§§^*p* < 0.01; ^§§§^*p* < 0.001 low-Na *vs* low-Na + CysA or FST

Total Cx40 protein was extracted from left atrial lysates by immunoprecipitation. Control experiments showed IP-Cx40 bound to Cx40-loaded beads, but unbound to Cx43-loaded beads. Total Cx40 (T-Cx40) was unaltered in low-Na solution (Fig. 5A, upper panels). However low-Na solution increased pSer-Cx40 and pThr-Cx40 expression. These actions of low-Na solutions were prevented by CAIP (upper panels). In addition, increase of pSer-Cx40 and pThr-Cx40 expression in low-Na solution were unaffected by TTM, but partially prevented by FST (Fig. 5A, lower panels).

Quantitative data for expression of pSer-Cx40 and pThr-Cx40 in low-Na solution, normalised to T-Cx40 levels, show an overall picture similar to that with pSer368-Cx43. Phosphorylation of pSer-Cx40 and pThr-Cx40 was low under control conditions (Fig. 5B,C) but was significantly increased in low-Na solution ((*p* < 0.001, *n* = 6). The raised pSer-Cx40 and pThr-Cx40 expression levels were partially prevented by CysA (*p* < 0.001, *n* = 6), and completely by CAIP. TTM had no significant effect on raised pSer-Cx40 and pThr-Cx40 expression in low Na-solution, but FST partially prevented the increase of pSer-Cx40 and pThr-Cx40 in low-Na solution (*p* < 0.001, *n* = 6).

## Discussion

### Raised [Ca^2+^]_i_, gap junction conductance and Cx43, Cx40 phosphorylation—the role of calcineurin

This study has shown that increased [Ca^2+^]_i_ in guinea-pig left atrial myocardium reduced gap junction electrical conductance (*G*_j_) by approximately 50%; and also increased Cx43 phosphorylation at serine368 (pSer368-Cx43), as well as Cx40 phosphorylation at serine and threonine residues. These changes were fully prevented by the selective calcineurin inhibitor CAIP, and partially so by the less selective agent cyclosporin-A. Moreover, changes were partly prevented by fostriecin, but unaffected by tautomycin suggesting an intermediate regulation of calcineurin action by the phosphatase PP2A, but none from PP1.

### Mode of action of Cn to regulate gap junction conductance

These observations differ in certain respects from equivalent experiments with guinea-pig ventricular myocardium. The basic observation that raised [Ca^2+^]_i_ reduces *G*_j_ and this is accompanied by increased ser368-Cx43 phosphorylation by a calcineurin-dependent pathway is common to both. However, calcineurin regulates this process in different ways. In the ventricle, calcineurin dephosphorylates an inhibitor of PP1 (I1) to activate PP1 itself and initiate ser365-Cx43 dephosphorylation; this in turn enables ser368-Cx43 phosphorylation [12]. In left atrial myocardium PP1 does not have an intermediary role for the action of calcineurin, as judged by the lack of action of its inhibitor tautomycin to prevent changes when [Ca^2+^]_i_ is raised. However, PP2A has at least a partial intermediary role as fostriecin partially prevented the changes to *G*_j_ and ser368-Cx43 phosphorylation induced by raising [Ca^2+^]_I_, this contrasts the lack of action of PP2A in ventricular myocardium [12]. This suggests the potential for differential regulation of gap junction electrical properties in atrial and ventricular myocardium when, for example, specific control of atrial or ventricular electrical disturbances is needed.

The additional role of Cx40 to determine *G*_j_ is a further differentiating feature of atrial myocardium. A similar increase of the phosphorylation of Cx40 serine and threonine residues was also mediated by a calcineurin-dependent pathway, with a partial role for PP2A, but none for PP1. However, it is unclear which particular phosphorylation sites on Cx40 regulate the conductance of gap junctions containing this subtype [19]. The significance of PP2A activity requires further study as this general name refers to a large family of proteins [20] and the particular subtype in guinea-pig needs to be referenced to that in human left atrium [21].^.^ However, a number of residues that alter activity may be phosphorylated, e.g. Tyr307-PP2A that decreases enzyme activity [22], and the role of calcineurin here requires evaluation.

**Fig. 6:**
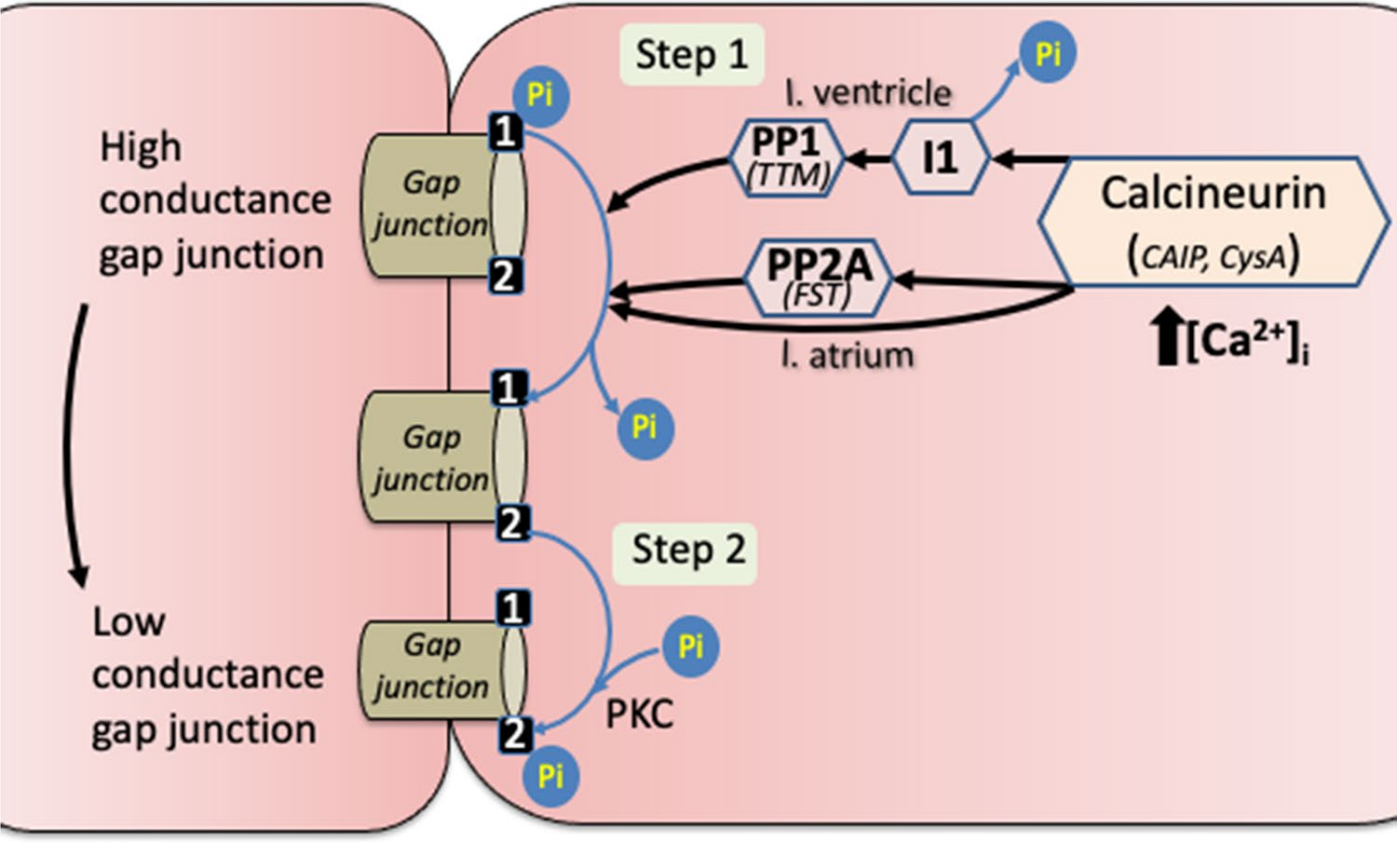
Regulation of myocardial gap junction electrical conductance (G_j_) by calcineurin. Gap junction (GJ) conductance is controlled by varying the phosphorylation of particular residues on connexin proteins (Cx43, Cx40) – two residues are shown (1, 2) one of which is phosphorylated (top GJ in diagram) to yield a high conductance channel. A rise of intracellular [Ca^2+^], [Ca^2+^]_i_, activates the phosphatase, calcineurin (Cn) to dephosphorylate residue-1 (step 1). This enables protein kinase-C (PKC) to phosphorylate residue-2, yielding a low conductance channel (step 2). Left atrial (LA) and left ventricular (LV) myocardium differ in the way Cn dephosphorylates residue-1. With LA tissue, data from this study are consistent with a direct action of Cn on residue-1, and partial intermediation of the phosphatase PP2A. With LV tissue, data (ref (12)) are consistent with dephosphorylation and deactivation of an inhibitor (I1) of the phosphatase PP1. Particular residues -1 and -2 on Cx43 are serine365 and serine368; but those on Cx40 involve unknown serine and threonine residues. The phosphatase inhibitors used are shown in italics under the relevant enzyme; see text for more details

Another potential Ca^2+^-regulated route to control *G*_j_ is by phosphorylation of Ser368-Cx43 by activation of CaMKII by Ca-calmodulin. However, this was deemed unlikely in left atrium as the CaMKII inhibitors KN-93 and AIP had no significant effect on *G*_j_ in low-Na solution. However, CaMKII could have an indirect role in sarcoplasmic reticulum Ca^2+^ dysregulation, further maintaining high [Ca^2+^]_i_ during AF [23]. By contrast, the PKC inhibitor chelerythrine prevented the reduction of *G*_j_ in low-Na solution and suggests that Cx43-Ser368 phosphorylation was mediated by PKC. A proposed scheme summarising the role of calcineurin in regulating the phosphorylation pattern of gap junction proteins is shown in Fig. 6.

### Calcineurin-dependent pathways and atrial fibrillation (AF)

Calcineurin-dependent pathways may influence the incidence and persistence of atrial arrhythmias by several routes. Firstly, calcineurin expression is greater in human left atrial samples from patients with AF [24], which could result from increased calpain activity, the up-stream activator of Cn [25], and/or up-regulation of *RCAN1* that transcribes a Cn regulator [26, 27].^.^ Secondly, reduction of *G*_j_, as shown in this study, will reduce the velocity of action potential propagation and contribute to emergence of re-entrant rhythms characteristic of AF. The importance of Ca^2+^/calcineurin-dependent pathways to determine Cx43 and Cx40 phosphorylation is an important feature. We showed previously a paradoxical age-dependent increase of Cx40 and Cx43 expression in human left atrium, despite an accompanying decrease of *G*_j_ [5].^.^ This further underlines the importance not of connexin expression *per se* but the extent of post-transcriptional modification as a crucial determinant of *G*_j_. Thirdly, calcineurin will affect ion channel current, either directly [28, 29] or by altering transcription of channel components. Calcineurin can exist either as CnAα or CnAβ, the former having rapid cytoplasmic actions, the latter acting as a chaperone for the transcription factor NFAT to enter the nucleus and initiate transcription [30, 31]. Overall calcineurin-dependent pathways can alter the electrical properties of myocardium either through long- or short-term routes: a short-term role during acute increase of intracellular [Ca^2+^] will importantly involve modification of gap-junction electrical properties.

### Changes to gap junction conductance and impact on action potential conduction velocity (CV)

The quantitative effect of the above changes to *G*_j_ on CV in atrial and ventricular myocardium may be estimated; in this case when intracellular [Ca^2+^]_i_ was raised – a high-Ca condition. This is relevant due to the pro-arrhythmic effects of reduced CV in a high-Ca state [12, 32]. CV is a positive function of total intracellular electrical conductance, (*G*_i_=(*G*_j·_*G*_c_)/(*G*_j_+*G*_c_)) – see also Table 1. One-dimensional cable theory shows that CV = *k*.√(*G*_i_), where *k* is a composite constant of other electrical properties of conducting myocardium [3, 33]. Thus, relative changes to *G*_i_ are determined not just by changes to *G*_j_, but also by their proportional relation to sarcoplasmic conductance, *G*_c_, which does not change under experimental conditions. Determination of atrial values of *G*_j_ and *G*_c_ reported here, and for ventricular myocardium in [5], give a unique opportunity to quantify more accurately the impact of altered gap junction properties on CV in these two tissues (Table 1).

**Table 1:** Experimental values (mS.cm^−1^) of specific gap junction (*G*_j_) and sarcoplasmic (*G*_c_) conductance in guinea-pig left atrial (*n* = 40) and ventricular (*n* = 20) myocardium in control and raised [Ca^2+^]_i_ (high-Ca) conditions

	*G*_j_ control	*G*_c_ control	*G*_j_ high-Ca	*G*_c_ high-Ca	*G*_i_ control	*G*_i_ high-Ca	CV, % control	CV, % high-Ca
Left atrium	8.61 ± 3.71	7.29 ± 2.17	5.05 ± 2.27	6.73 ± 2.45	3.95 ± 0.52	2.89 ± 0.35	100	85.5 ± 5.2
Left ventricle	3.80 ± 0.70 *	5.94 ± 3.09	2.00 ± 0.39 *	5.65 ± 1.01	2.32 ± 0.65 *	1.48 ± 0.48 *	100	79.9 ± 3.0 *

Total intracellular conductance, *G*_i_, was calculated as: *G*_i_ = (*G*_j_.*G*_c_)/(*G*_j_ + *G*_c_). Conduction velocity in high-Ca is expressed as a percentage of the control values. *p < 0.05 ventricular *vs* atrial values

The data show that gap junction conductance, *G*_j_, is significantly greater in atrial than in ventricular myocardium in both control and high-Ca conditions, but sarcoplasmic conductance, *G*_c_, is similar in both tissues and under control and high-Ca conditions. In consequence, total intracellular conductance, *G*_i_, is greater in atrial myocardium in both control and high-Ca conditions. The larger value of *G*_i_ in atrial myocardium is consistent with a greater CV compared to ventricular myocardium [3]. In ventricular myocardium a reduction of *G*_j_ by about 50% (i.e. [12]) is estimated to reduce CV by about 20%, if all other biophysical characteristics [3, 33] remain constant. However, in atrial myocardium a similar proportional reduction of *G*_j_ would have a significantly smaller effect on CV, due to the greater proportional impact that *G*_c_ has on total intracellular conductance. Thus, a similar impact on atrial gap junction electrical properties would have a smaller consequence on CV, compared with ventricular myocardium.

### Limitations

1. Avoidance of signal drift with impedance measurements is important to compare effects of interventions with controls. Each experiment included short-term and long-term time controls: A short-term control was two ‘impedance runs’, five minutes apart for each intervention: data were not used if the difference exceeded 5%. A long-term control was inclusion of a second Tyrode’s intervention after exposure to low-Na solution. Data from preparations were also not used if *G*_j_ values differed by more than 10%: in total such drift occurred is four of 44 preparations.
2. Vehicle controls were not included to minimise use of animals. In particular, stock solutions of CysA, FST and KN-93 were dissolved in DMSO, with final solvent concentrations of 0.05, 0.0005 and 0.01% (v/v) respectively. Other work has shown DMSO to affect cell membrane biophysical properties only above 0.5–1% concentration [34].

## Conclusions

Calcineurin-dependent phosphorylation of connexin proteins (Cx40 and Cx43) is a key determinant of gap junction conductance and hence action potential propagation in left atrial myocardium. This particular calcineurin-dependent pathway, with the partial involvement of PP2A, is different in left atrium compared to ventricular myocardium and raises the possibility of targeted anti-arrhythmic therapies aimed more specifically at the two chambers.

## Supplementary Information

Below is the link to the electronic supplementary material.

ESM 1 (PNG 912 kb)

High resolution image (TIFF 1142 kb)

## Data Availability

Reasonable requests for data will be considered by Prof Christopher Fry.

## Abbreviations, acronyms and terms

AF: Atrial fibrillation
AIP: Autocamtide-2-Related Inhibitory Peptide, CaMKII inhibitor
AP: Action potential
CAIP: Calcineurin auto-inhibitory peptide
Chelerythrine: PKC inhibitor
Cn: Calcineurin
Cx: Connexin
CaMKII: Calcium-calmodulin dependent protein kinase II
pSer368-Cx43: Phosphorylated Cx43 at serine 368
CysA: Cyclosporin-A, Cn inhibitor
FST: Fostriecin, PP2A inhibitor
G_c_: Specific sarcoplasmic (cytoplasmic) conductance
G_i_: Specific total intracellular conductance
G_j_: Specific gap junction conductance
I1: Inhibitor 1 (of PP1)
IP: Immunoprecipitation
KN-93: CaMKII inhibitor
LA: Left atrium
mS: MilliSiemen, unit of electrical conductance
PKC: Protein kinase C
PP: Protein phosphatase
PP1: Protein phosphatase 1
PP2A: Protein phosphatase 2A
Ser: Serine
‘specific’: Cellular electrical constants expressed independent of cell/tissue dimensions
Thr: Threonine
TTM: Tautomycin, PP1 inhibitor
