# Supplementary figures and images for: Calcineurin-dependent regulation of gap junction conductance and connexin phosphorylation in guinea pig left atrium
Source: Pflugers Arch. 2023 Mar 14;475(5):583–93. doi: 10.1007/s00424-023-02798-9 (PMC10105670; doi:10.1007/s00424-023-02798-9)

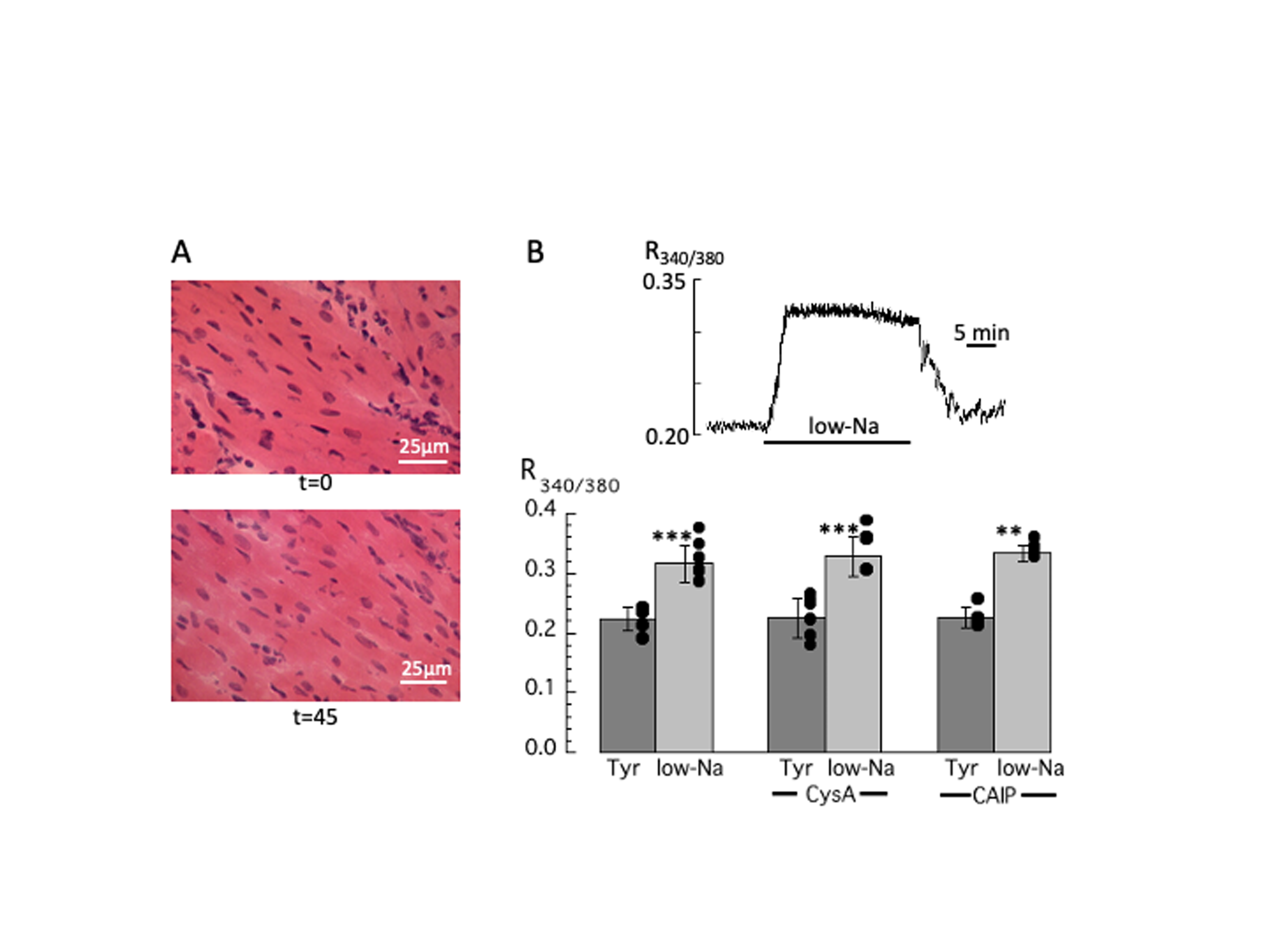

Supplement: Supplementary file 1 — (PNG 912 kb) [file 424_2023_2798_Fig7_ESM.png]

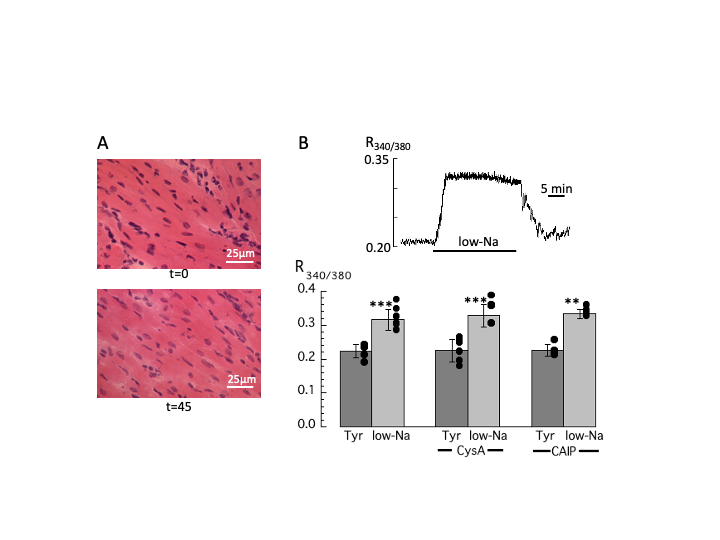

Supplement: Supplementary file 2 — High resolution image (TIFF 1142 kb) [file 424_2023_2798_MOESM1_ESM.tiff]
